# Prognostic factors and survival outcomes in older adults with extensive-stage small cell lung cancer: A multicenter retrospective cohort study

**DOI:** 10.1097/MD.0000000000048711

**Published:** 2026-05-08

**Authors:** Sermin Dinc Sonusen, Zehra Sucuoglu Isleyen, Naime Afsar Satis, Mesut Yilmaz, Bekir Dogan, Senar Gunenc, Tanju Berber, Berna Akkus Yildirim, Yaren Ceran Bas, Merve Ekinci Fidan, Resit Akyel, Kayhan Erturk, Emir Celik, Muhammed Mustafa Atci

**Affiliations:** a Department of Medical Oncology, Prof. Dr. Cemil Tascioglu City Hospital, Istanbul, Turkey; b Department of Medical Oncology, Bakirkoy Dr Sadi Konuk Training and Research Hospital, Istanbul, Turkey; c Department of Radiation Oncology, Yedikule Chest Diseases and Chest Surgery Training and Research Hospital, Istanbul, Turkey; d Department of Radiation Oncology, Prof. Dr. Cemil Tascioglu City Hospital, Istanbul, Turkey; e Department of Thoracic Surgery, Yedikule Chest Diseases and Chest Surgery Training and Research Hospital, Istanbul, Turkey; f Department of Nuclear Medicine, Yedikule Chest Diseases and Chest Surgery Training and Research Hospital, Istanbul, Turkey.

**Keywords:** extensive stage, small cell lung cancer, survival and prognosis

## Abstract

Extensive-stage small cell lung cancer (ES-SCLC) in older adults is associated with poor outcomes, and optimal management in this population remains challenging. This study aimed to identify clinical and treatment-related factors associated with survival in patients aged ≥70 years with ES-SCLC. A retrospective multicenter cohort study included 135 older adults (aged ≥70 years) diagnosed with ES-SCLC across 3 tertiary centers. All patients received at least 1 cycle of platinum–etoposide–based chemotherapy. Demographic, clinical, and treatment variables – including metastatic sites, number of chemotherapy cycles, thoracic radiotherapy (TRT), prophylactic cranial irradiation, and palliative radiotherapy to extracranial metastases – were collected. Survival outcomes were estimated using the Kaplan–Meier method and compared using the log-rank test. Prognostic factors for progression-free survival and overall survival (OS) were evaluated using Cox proportional hazards regression models. The median age was 73.2 years; 88.1% of patients were male. The objective response rate was 69.9%. Median progression-free survival and OS were 7.1 and 8.3 months, respectively (95% CI: 6.09–8.17 and 7.67–8.93). In multivariate analysis, Eastern Cooperative Oncology Group performance status ≥2 (*P* <.001), liver metastases (*P* = .006), fewer than 4 chemotherapy cycles (*P* <.001), absence of prophylactic cranial irradiation (*P* = .028), and lack of palliative radiotherapy to extracranial metastases (*P* <.001) were independently associated with shorter OS. TRT did not retain statistical significance in multivariate analysis. Only 34.1% of patients received second-line therapy. The 1-year and 2-year OS rates were 33% and 12%, respectively. Fit older adults (Eastern Cooperative Oncology Group performance status 0–1) may derive meaningful survival benefit from multimodal treatment strategies. Treatment decisions should be individualized based on functional status rather than chronological age alone. Prospective studies incorporating comprehensive geriatric assessment are warranted.

## 1. Introduction

Small cell lung cancer (SCLC) accounts for approximately 15% of all lung cancers and is characterized by rapid tumor growth and early metastatic dissemination, with most patients presenting with extensive-stage (ES) disease at diagnosis.^[1,2]^ ES-SCLC is associated with aggressive clinical behavior and poor survival outcomes despite recent therapeutic advances.^[2,3]^

Platinum–etoposide chemotherapy has long been the standard first-line treatment for ES-SCLC. The addition of programmed death-ligand 1 (PD-L1) inhibitors, including atezolizumab and durvalumab, to platinum–etoposide chemotherapy has demonstrated statistically significant improvements in overall survival and has established chemoimmunotherapy as the current standard of care in the first-line setting.^[4–7]^

In selected patients who respond to systemic therapy, consolidative thoracic radiotherapy (TRT) has been shown to improve intrathoracic disease control and may provide a survival benefit.^[8]^ Prophylactic cranial irradiation (PCI) has been shown to reduce the incidence of brain metastases and improve overall survival in earlier randomized trials of ES-SCLC.^[9]^ However, subsequent prospective data have yielded conflicting results, particularly in the context of routine brain magnetic resonance imaging surveillance.^[10]^ Moreover, concerns about neurocognitive toxicity and the evolving therapeutic landscape in the immunotherapy era have intensified the debate regarding the routine use of PCI in ES-SCLC. In patients with established brain metastases, whole-brain radiotherapy (WBRT) or stereotactic radiosurgery (SRS) remain standard treatment approaches, while palliative radiotherapy continues to play an important role in symptom control.^[11–13]^

Despite these therapeutic advances, ES-SCLC remains an aggressive disease with a high risk of rapid progression and limited survival. With increasing global life expectancy, the proportion of older adults diagnosed with cancer is rising, and lung cancer remains a leading cause of cancer-related mortality in this population.^[14]^ In older adults, comorbidities, frailty, and impaired performance status (PS) frequently complicate treatment decision-making, underscoring the need to identify prognostic factors that may inform individualized therapeutic strategies. Although several studies have evaluated prognostic factors in SCLC, most analyses have included heterogeneous age populations, and older adults remain underrepresented in pivotal chemoimmunotherapy trials. Moreover, limited data are available specifically addressing clinical outcomes and prognostic determinants in patients aged ≥ 70 years with ES-SCLC in the contemporary treatment era. Therefore, this multicenter retrospective cohort study aimed to evaluate clinical and treatment-related prognostic factors associated with survival in patients aged ≥70 years with ES-SCLC, a population that remains underrepresented in prospective clinical trials.

## 2. Methods

### 2.1. Study design

This retrospective multicenter cohort study was conducted at Prof Dr Cemil Tascioglu City Hospital, Yedikule Chest Diseases and Chest Surgery Training and Research Hospital, and Bakirkoy Dr Sadi Konuk Training and Research Hospital in Istanbul, Turkey. The study included patients aged ≥70 years diagnosed with ES SCLC (ES-SCLC) between January 2009 and December 2024. Clinical data were retrieved from electronic medical records.

Ethical approval was obtained from the Prof Dr Cemil Tascioglu City Hospital Ethics Committee (Approval No: 204, August 19, 2025). The study was conducted in accordance with the principles of the Declaration of Helsinki. This study was reported in compliance with the Strengthening the Reporting of Observational Studies in Epidemiology (STROBE) guidelines.^[15]^

### 2.2. Participants

Eligibility criteria comprised histopathologically confirmed ES-SCLC, receipt of at least 1 cycle of systemic chemotherapy, and availability of complete medical and survival data. Patients with incomplete records or lost to follow-up after initial treatment were excluded.

Tumor staging was performed using the Veterans Administration Lung Study Group 2-stage classification system.^[16]^ PS was assessed according to the Eastern Cooperative Oncology Group (ECOG) criteria.^[17]^

### 2.3. Assessments

Demographic, clinical, and treatment-related data – including metastasis patterns, chemotherapy regimens, radiotherapy details, and comorbidities – were extracted from electronic medical records.

All patients received first-line chemotherapy with cisplatin or carboplatin combined with etoposide, administered according to institutional standards and contemporary international guidelines.^[4,5]^ A small subset received oral etoposide alone, and a limited number were treated with carboplatin–etoposide–atezolizumab combination therapy.^[4,5]^

Data on the number of chemotherapy cycles, use of consolidative TRT, PCI, palliative radiotherapy to extracranial metastases, and cranial radiotherapy for brain metastases were recorded.

Tumor response was assessed radiologically following first-line therapy completion according to RECIST version 1.1 criteria and categorized as complete response (CR), partial response (PR), stable disease (SD), or progressive disease.^[18]^ objective response rate was defined as the sum of CR and PR rates.

Progression-free survival (PFS) was defined as the time from first-line chemotherapy initiation to disease progression or death from any cause, whichever occurred first. Overall survival (OS) was measured from diagnosis to death or last follow-up.

## 3. Statistical analysis

Descriptive statistics were used to summarize demographic and clinical characteristics. Categorical variables were expressed as frequencies and percentages. Continuous variables were presented as mean ± standard deviation or median (interquartile range, IQR), as appropriate. The normality of continuous variables was assessed using the Kolmogorov–Smirnov test. Variables that did not show normal distribution were analyzed using nonparametric methods. PFS and OS were estimated using the Kaplan–Meier method, and survival curves were compared using the log-rank test. The Breslow (generalized Wilcoxon) test was additionally applied to evaluate early survival differences. Univariate and multivariate Cox proportional hazards regression analyses were performed to identify factors associated with PFS and OS. Variables with *P* <.20 in univariate analysis were included in the multivariate models. hazard ratios and 95% confidence intervals were reported. All statistical analyses were conducted using IBM SPSS Statistics for Windows, Version 26.0 (IBM Corp., Armonk). A 2-sided *P*-value <.05 was considered statistically significant.

## 4. Results

A total of 135 patients with ES-SCLC were included. The median age was 73.2 years, with 88.1% (n = 119) male and 11.9% (n = 16) female. Baseline demographics and clinical characteristics are summarized in Table 1.

**Table 1:** Baseline demographic and clinical characteristics of the study population.

Characteristics	n:135	%
Sex		
Women	16	11.9
Men	119	88.1
ECOG PS		
0–1	94	69.6
≥2	41	30.4
Contralateral lung metastasis		
No	97	71.9
Yes	38	28.1
Pleura metastasis		
No	95	70.4
Yes	40	29.6
Liver metastasis		
No	92	68.1
Yes	43	31.9
Adrenal metastasis		
No	113	83.7
Yes	22	16.3
Brain metastasis		
No	106	78.5
Yes	29	21.5
Bone metastasis		
No	62	45.9
Yes	73	54.1
Extrathoracic lymph node metastasis		
No	51	38.1
Yes	83	61.9
First-line treatment		
Cisplatin + etoposide	65	48.1
Carboplatin + etoposide	56	41.5
Oral etoposide	8	5.9
Carboplatin + etoposide + atezolizumab	6	4.4
Number of cycles in the first-line		
<4	37	27.4
≥4	98	72.6
Radiologic response assessment available		
No	19	14.1
Yes	116	85.9
Response to first-line treatment		
CR	27	23.3
PR	54	46.6
SD	18	15.5
PD	17	14.7
Second-line treatment		
No	89	65.9
Yes	46	34.1
Irradiation of the thoracic residual disease		
No	64	47.4
Yes	71	52.6
Prophylactic cranial irradiation		
No	106	78.5
Yes	29	21.5
Cranial radiotherapy for brain metastases		
No	105	77.8
WBRT	24	17.8
SRS	6	4.4
Radiotherapy to extracranial sites		
No	92	68.1
Yes	43	31.9
Comorbidities		
Hypertension	87	64.4
COPD	77	57.0
Hyperlipidemia	42	31.1
Diabetes mellitus	40	29.6
Coronary artery disease	37	27.4
Benign prostatic hyperplasia	11	8.1
Congestive heart failure	8	5.9
Parkinson disease	5	3.7
Ischemic cerebrovascular disease	3	2.2

% = percentage, COPD = chronic obstructive pulmonary disease, CR = complete response, ECOG PS = Eastern Cooperative Oncology Group performance status, n = number of patients, PD = progressive disease, PR = partial response, SD = stable disease, SRS = stereotactic radiosurgery, WBRT = whole-brain radiotherapy.

Of the cohort, 35 patients received systemic therapy alone (chemotherapy or chemoimmunotherapy) without radiotherapy. Consolidative TRT was administered to 71 patients (52.6%), and PCI to 29 (21.5%) following chemotherapy. Three patients undergoing PCI and 4 patients receiving TRT had SD at the time of irradiation. Cranial radiotherapy (WBRT or SRS) was performed in 30 patients (22.2%) for brain metastases at any treatment stage. Palliative radiotherapy for extracranial metastases was delivered to 43 patients (31.9%). Regarding chemotherapy, 72.6% received 4 or more cycles, whereas 27.4% received fewer than 4. Only 6 patients were treated with the carboplatin, etoposide, and atezolizumab combination. After progression, 34.1% received second-line therapy.

Radiological response assessment post first-line chemotherapy was available for 116 patients (85.9%). The objective response rate was 69.9%, comprising 23.3% CR and 46.6% PR. SD and progressive disease were observed in 15.5% and 14.7% of patients, respectively.

Median PFS was 7.1 months (95% CI: 6.09–8.17), and median OS was 8.3 months (95% CI: 7.67–8.93). Disease progression occurred in 123 patients. PFS rates at 6, 12, 24, 36, and 48 months were 58%, 18%, 6%, 3%, and 1%, respectively.

Multivariate analysis identified poor ECOG PS (ECOG PS ≥2; *P* <.001), liver metastases (*P* = .028), fewer than 4 chemotherapy cycles (*P* <.001), absence of PCI (*P* = .019), and lack of palliative radiotherapy to extracranial metastases (*P* = .020) as independent predictors of shorter PFS (Table 2).

**Table 2:** Univariate and multivariate cox regression analyses for progression-free survival.

	Univariate analysis			Multivariate analysis		
Variables	HR	95 % CI	*P*-value	HR	95 % CI	*P*-value
Sex						
Women (Ref)	0.96	0.56–1.63	.868	–	–	–
Men						
ECOG PS						
0–1 (Ref)	3.00	2.01–4.49	<.001	2.64	1.70–4.11	<.001
≥2						
Age	1.08	1.03–1.14	.002	1.02	0.96–1.08	.494
Location						
Right (Ref)	1.22	0.86–1.75	.265	–	–	–
Left						
Pleura metastasis						
No (Ref)	1.24	0.84–1.83	.267	–	–	–
Yes						
Contralateral lobe metastasis						
No (Ref)	0.92	0.62–1.37	.685	–	–	–
Yes						
Liver metastasis						
No (Ref)	1.54	1.04–2.30	.032	1.58	1.05–2.39	.028
Yes						
Adrenal metastasis						
No (Ref)	1.15	0.71–1.84	.572	–	–	–
Yes						
Bone metastasis						
No (Ref)	0.86	0.60–1.24	.423	–	–	–
Yes						
Cranial metastasis						
No (Ref)	1.78	1.14–2.78	.012	0.76	0.46–1.25	.273
Yes						
Lymph node metastasis						
No (Ref)	0.86	0.59–1.23	.403	–	–	–
Yes						
Number of metastatic sites						
Single site of metastasis (Ref)	0.90	0.60–1.36	.627	–	–	–
>1 site of metastasis						
First-line treatment						
Cisplatin-Etoposide (Ref)	–	–	.011	–	–	.976
Carboplatin–Etoposide	0.97	0.66–1.43	.885	1.01	066–1.55	.968
Etoposide	3.24	1.54–6.83	.002	1.19	0.50–2.83	.684
Carboplatin-Etoposide–Atezolizumab	0.69	0.28–1.73	.432	0.91	0.35–2.37	.853
Number of cycles in the first-line						
≥4 (Ref)	5.91	3.85–9.08	<.001	4.76	2.99–7.58	<.001
<4						
Irradiation of the thoracic residual disease						
Yes (Ref)	1.72	1.21–2.47	.003	1.16	0.77–1.75	.484
No						
Prophylactic cranial irradiation						
Yes (Ref)	2.40	1.52–3.79	<.001	1.79	1.10–2.92	.019
No						
Radiotherapy to extracranial sites						
Yes (Ref)	1.65	1.11–2.45	.013	1.66	1.08–2.53	.020
No						

% = percentage, CI = confidence interval, ECOG PS = Eastern Cooperative Oncology Group performance status, HR = hazard ratio.

At analysis, 121 patients had died. OS rates at 6, 12, 24, 36, and 48 months were 69%, 33%, 12%, 6%, and 4%, respectively. Independent predictors of worse OS were similar to those identified for PFS: ECOG PS ≥2 (*P* <.001), liver metastases (*P* = .006), fewer chemotherapy cycles (*P* <.001), absence of PCI (*P* = .028), and no palliative radiotherapy (*P* <.001) (Table 3).

**Table 3:** Univariate and multivariate cox regression analyses for overall survival.

	Univariate analysis			Multivariate analysis		
Variables	HR	95 % CI	*P*-value	HR	95 % CI	*P*-value
Sex						
Women (Ref)	1.01	0.58–1.72	.998	–	–	–
Men						
ECOG PS						
0–1 (Ref)	3.54	2.36–5.29	<.001	3.03	1.92–4.81	<.001
≥2						
Age	1.11	1.06–1.17	<.001	1.06	0.99–1.12	.055
Location						
Right (Ref)	1.16	0.81–1.66	.431	–	–	–
Left						
Pleura metastasis						
No (Ref)	1.15	0.78–1.71	.486	–	–	–
Yes						
Contralateral lobe metastasis						
No (Ref)	1.03	0.69–1.53	.888	–	–	–
Yes						
Liver metastasis						
No (Ref)	1.80	1.21–2.69	.004	1.81	1.19–2.76	.006
Yes						
Adrenal metastasis						
No (Ref)	1.26	0.79–2.01	.323	–	–	–
Yes						
Bone metastasis						
No (Ref)	0.79	0.55–1.13	.194	0.87	0.59–1.28	.475
Yes						
Cranial metastasis						
No (Ref)	2.14	1.36–3.35	.001	1.01	0.61–1.66	.981
Yes						
Lymph node metastasis						
No (Ref)	0.73	0.50–1.06	.099	1.17	0.75–1.84	.494
Yes						
Number of metastatic sites						
Single site of metastasis	0.89	0.59–1.35	.586	–	–	–
>1 site of metastasis						
First-line treatment						
Cisplatin-Etoposide (Ref)	–	–	.003	–	–	.556
Carboplatin-Etoposide	0.90	0.61–1.32	.592	0.80	0.52–1.25	.331
Etoposide	4.22	1.88–9.42	<.001	1.39	0.60–3.20	.444
Carboplatin-Etoposide-Atezolizumab	0.83	0.33–2.07	.689	0.80	0.32–2.04	.647
Number of cycles in the first-line						
≥4 (Ref)	6.24	4.01–9.69	<.001	5.29	3.27–8.56	<.001
<4						
Irradiation of the thoracic residual disease						
Yes (Ref)	1.84	1.28–2.65	.001	1.15	0.73–1.81	.549
No						
Prophylactic cranial irradiation						
Yes (Ref)	2.14	1.35–3.39	.001	1.76	1.06–2.90	.028
No						
Radiotherapy to extracranial sites						
Yes (Ref)	2.16	1.24–3.25	<.001	2.52	1.61–3.94	<.001
No						

% = percentage, CI = confidence interval, ECOG PS = Eastern Cooperative Oncology Group performance status, HR = hazard ratio.

**Figure 1.**
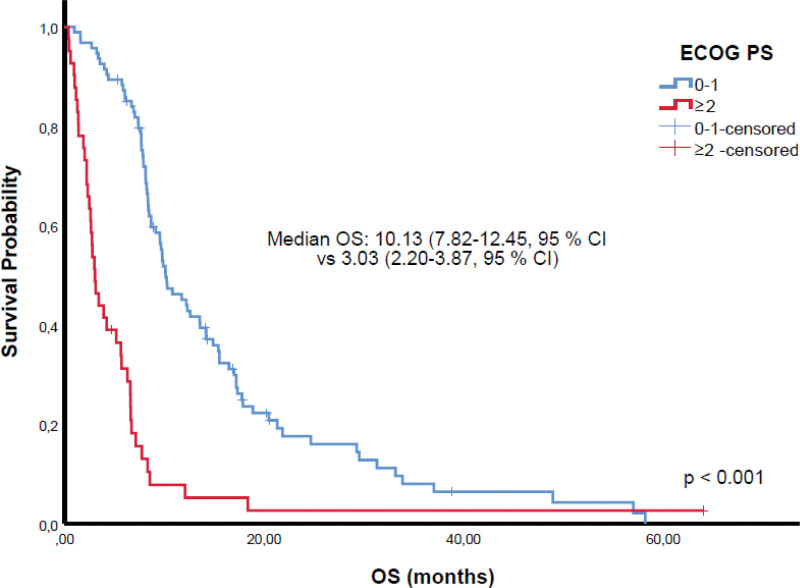
Overall survival stratified by ECOG performance status; patients with ECOG PS 0–1 had significantly longer overall survival compared with those with ECOG PS ≥2. CI = confidence interval, ECOG PS = Eastern Cooperative Oncology Group performance status, OS = overall survival.

**Figure 2.**
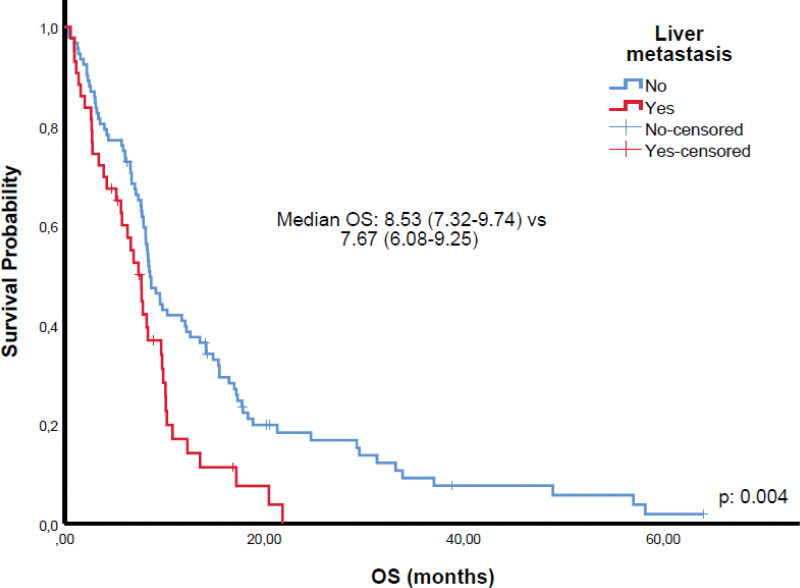
Overall survival according to liver metastasis status; absence of liver metastases was associated with improved overall survival. OS = overall survival.

**Figure 3.**
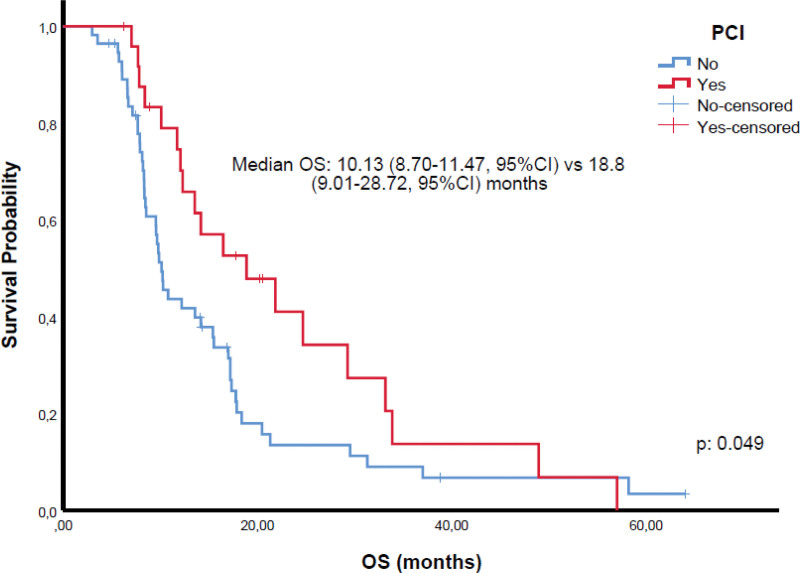
Overall survival among responders (CR/PR) to first-line therapy, stratified by prophylactic cranial irradiation; receipt of PCI was associated with improved overall survival. CI = confidence interval, CR = complete response, OS = overall survival, PCI = prophylactic cranial irradiation, PR = partial response.

**Figure 4.**
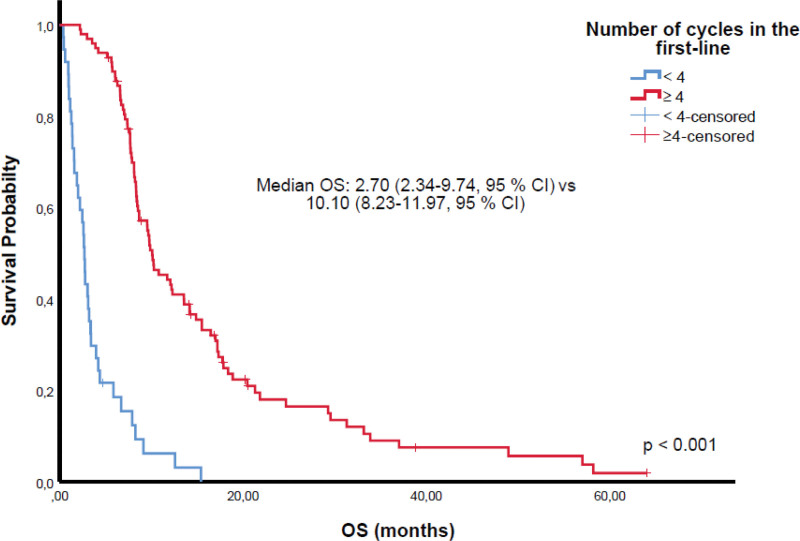
Overall survival according to the number of chemotherapy cycles; receipt of ≥4 cycles was associated with superior overall survival compared with < 4 cycles. CI = confidence interval, OS = overall survival.

Among 81 patients achieving CR or PR, 67 received TRT and 25 PCI. While TRT was associated with survival benefit in univariate analysis, this was not sustained in multivariate models (Tables 2 and 3). Kaplan–Meier analyses demonstrated significantly shorter OS in patients with poor ECOG PS (Fig. 1) and liver metastases (Fig. 2). Patients with CR/PR who underwent PCI had improved OS compared to those without PCI (Fig. 3). Furthermore, receiving ≥4 chemotherapy cycles correlated with longer OS (Fig. 4). Subgroup analysis of immunotherapy-treated patients was not performed due to limited sample size.

## 5. Discussion

In this multicenter retrospective cohort study of patients aged ≥70 years with ES-SCLC, poor ECOG PS, liver metastases, completion of fewer than 4 chemotherapy cycles, absence of PCI, and lack of extracranial palliative radiotherapy were identified as independent predictors of inferior progression-free and overall survival. Despite advances in systemic therapy, survival outcomes in this older adult population remain limited. The median overall survival of 8.3 months observed in our cohort is consistent with previously reported outcomes in older patients with ES-SCLC.^[19,20]^ The age threshold of ≥70 years was selected to reflect a clinically meaningful older adult population that remains underrepresented in prospective clinical trials.

Management of older adults with ES-SCLC remains challenging due to high comorbidity burden, frailty, and treatment-related toxicity risks. Advanced age and high cumulative tobacco exposure predispose this population to pulmonary and cardiovascular diseases, polypharmacy, and consequently, reduced treatment tolerance. Previous studies, such as Stinchcombe et al, have documented inferior survival and treatment completion rates, as well as higher rates of severe adverse events, in older patients.^[21]^ Similarly, large trials like IMpower133 reported median OS of approximately 9.6 months in patients over 65 years, paralleling findings from other cohorts with older adult subgroups.^[6,19]^ Barriers including toxicity concerns, limited caregiver support, and financial constraints often limit access to optimal therapy, as highlighted by Schild et al, where 30% of patients aged ≥80 years remained untreated.^[22]^ Even access to palliative care was limited for older adult patients.^[23]^

Platinum–etoposide chemotherapy remains the backbone of ES-SCLC treatment, with emerging data supporting the addition of immune checkpoint inhibitors like atezolizumab or durvalumab, which have extended median OS beyond 1 year in clinical trials.^[6,7]^ However, in our cohort, immunotherapy use was scarce due to limited reimbursement and accessibility during the study period. Retrospective analyses suggest comparable survival between older adults and younger patients receiving chemoimmunotherapy, without increased toxicity, underscoring the potential for expanding such treatments in older adults.^[24,25]^

Our multivariate analysis identified poor ECOG PS (≥2), liver metastases, fewer than 4 cycles of chemotherapy, omission of PCI, and absence of extracranial palliative radiotherapy as independent predictors of inferior PFS and OS. The prognostic significance of ECOG PS aligns with existing literature, reflecting its influence on treatment tolerance and ability to complete planned therapy.^[19,22]^ Patients with ECOG PS ≥2 are less likely to receive aggressive multimodal therapy, contributing to poorer outcomes.

Liver metastasis emerged as a robust negative prognostic factor, consistent with its association with aggressive tumor biology and systemic dissemination.^[4,5,26]^ Completion of at least 4 chemotherapy cycles conferred a significant survival advantage, highlighting the importance of treatment completion when clinically feasible, even in older patients. Early treatment discontinuation likely reflects frailty, comorbidities, or aggressive disease limiting tolerability and efficacy.

The role of PCI remains debated. The EORTC trial and several studies demonstrated OS benefit and reduced brain metastases incidence.^[9,27,28]^ The Japanese study by Takahashi et al did not confirm survival improvement with PCI in the context of regular magnetic resonance imaging surveillance.^[10]^ Our findings suggest a potential benefit of PCI in extending OS in older adults, particularly in settings where frequent neuroimaging is unavailable.

Consolidative TRT was associated with improved survival in univariate analysis but did not retain significance in multivariate models. This discrepancy may stem from limited sample size and heterogeneity in treatment application. Nonetheless, TRT remains an important consideration for selected older adults with controlled extrathoracic disease and good ECOG PS.^[8,29]^

Limitations of our study include its retrospective design, missing data on frailty scores and treatment toxicity, and a small number of patients receiving immunotherapy due to reimbursement and socioeconomic constraints. In addition, the limited use of immunotherapy restricts the generalizability of our findings to the contemporary chemoimmunotherapy era. The observed association between absence of extracranial radiotherapy and poorer survival likely reflects both therapeutic benefit in oligometastatic patients and selection bias, emphasizing the potential value of aggressive local therapy even in ES-SCLC, warranting prospective validation.

As immunotherapy may alter the natural history of brain metastases, the role of PCI in the chemoimmunotherapy era requires reevaluation through future prospective trials.^[30]^ Additionally, dedicated research incorporating geriatric assessments, quality of life measures, and treatment tolerability is essential to develop personalized management strategies for older adults with ES-SCLC. Given the increasing number of older adults with cancer globally, age-adapted evidence-based approaches are urgently needed.

## 6. Conclusion

Completion of ≥4 chemotherapy cycles, receipt of PCI, and administration of extracranial palliative radiotherapy were independently associated with improved survival outcomes. Consolidative thoracic radiotherapy demonstrated benefit in univariate analysis but was not an independent predictor after multivariable adjustment. Poor ECOG PS and liver metastases were independently associated with worse survival.

These findings suggest that selected fit older adults may benefit from a multimodal treatment approach, and treatment decisions should not be based solely on chronological age. Given the retrospective design of the study, prospective trials incorporating comprehensive geriatric assessments are warranted to better define optimal treatment strategies in this population.

## Author contributions

**Conceptualization:** Sermin Dinc Sonusen, Zehra Sucuoglu Isleyen, Muhammed Mustafa Atci.

**Data curation:** Naime Afsar Satis, Mesut Yilmaz, Bekir Dogan, Senar Gunenc, Tanju Berber, Yaren Ceran Bas.

**Formal analysis:** Sermin Dinc Sonusen, Zehra Sucuoglu Isleyen.

**Investigation:** Sermin Dinc Sonusen, Zehra Sucuoglu Isleyen, Naime Afsar Satis, Mesut Yilmaz, Bekir Dogan, Senar Gunenc, Berna Akkus Yildirim, Tanju Berber, Yaren Ceran Bas.

**Methodology:** Mesut Yilmaz, Berna Akkus Yildirim, Kayhan Erturk, Emir Celik, Muhammed Mustafa Atci.

**Project administration:** Kayhan Erturk, Emir Celik, Muhammed Mustafa Atci.

**Supervision:** Kayhan Erturk, Emir Celik, Muhammed Mustafa Atci.

**Visualization:** Sermin Dinc Sonusen, Zehra Sucuoglu Isleyen.

**Writing – original draft:** Sermin Dinc Sonusen, Merve Ekinci Fidan, Resit Akyel.

**Writing – review & editing:** Sermin Dinc Sonusen, Zehra Sucuoglu Isleyen, Kayhan Erturk, Muhammed Mustafa Atci.
